# Bovine lactoferrin maintains antibacterial effect against neonatal Escherichia coli septicaemia isolates despite the presence of iron acquisition genes

**DOI:** 10.1099/jmm.0.002116

**Published:** 2026-01-21

**Authors:** Matthew Mayorga, Joshua L. Wheatley, Brianna N. Maldonado, Susana Chavez-Bueno

**Affiliations:** 1 Kansas City University College of Osteopathic Medicine, Kansas City, MO, USA; 2 Division of Infectious Diseases, Children’s Mercy Kansas City, Kansas City, MO, USA; 3 University of Missouri Kansas City School of Medicine, Kansas City, MO, USA

**Keywords:** antibiotic resistance, *Escherichia coli*, lactoferrin, neonatal sepsis, virulence, whole-genome sequencing

## Abstract

**Introduction.** *Escherichia coli* is a dominant cause of neonatal sepsis for which no prevention measures currently exist. Lactoferrin (LF) is an iron-sequestering antibacterial protein that has been noticed to decrease neonatal late-onset sepsis.

**Hypothesis/Gap Statement.** LF’s effect on *in vitro* growth of neonatal *E. coli* clinical isolates causing septicaemia is unknown. The prevalence of iron acquisition virulence genes which contribute to pathogenicity outcomes in these strains has also not been described in detail.

**Aim.** To evaluate bovine lactoferrin’s (bLF) effects on the *in vitro* growth of neonatal septicaemia *E. coli* isolates, and to determine the presence of iron acquisition virulence genes in these isolates.

**Methodology.** *E. coli* clinical strains isolated from blood of septic neonates, including the archetypal RS218 strain, all representing prevalent phylogroups and multi-locus sequence types among neonatal invasive strains were studied. Strains were grown in batch growth with 0, 0.1, 1.0 and 10 mg ml^−1^ bLF. OD measurements over time were used to generate growth curves, and the area under these curves was statistically compared using ANOVA or Kruskal–Wallis tests. Whole-genome sequencing (WGS) data were used to identify iron acquisition genes.

**Results.** For all strains, growth decrease with 10 mg ml^−1^ bLF compared to zero was highly significant (*P*<0.001), and with 1 mg ml^−1^ for some strains (*P*≤0.02). WGS showed that all neonatal strains carried several iron acquisition virulence genes, including *chuA*, *fyuA*, *irp2* and *sitA*. Additional siderophore genes were found in some strains.

**Conclusion.** bLF is effective in impairing growth of neonatal sepsis-causing *E. coli* regardless of the presence of multiple iron acquisition virulence genes.

**Impact Statement**

This study investigates the impact of bovine lactoferrin (bLF), a widely available antibacterial protein, on the growth of *Escherichia coli* strains that cause neonatal septicaemia, and explores the presence of iron acquisition virulence genes in these strains. *E. coli* is a leading cause of neonatal sepsis, and no preventative measures exist. Neonatal *E. coli* isolates representing various phylogenetic backgrounds among invasive strains that caused sepsis in newborns were studied. Isolates were cultured in various concentrations of bLF, and growth was assessed over time. All strains demonstrated statistically significant growth suppression, even though they possessed iron acquisition virulence genes. This study provides novel data on the concentration-dependent antibacterial effect of bLF against neonatal *E. coli* septicaemia isolates, which has not been evaluated before, particularly in the current era of increased antibiotic resistance among these invasive strains. The results of this work support the role of bLF as a potential adjunct or alternative to standard treatments against neonatal *E. coli* sepsis, thus increasing the knowledge on the role of bLF as an effective antimicrobial countermeasure. In addition, the antibacterial effects obtained at the bLF concentrations tested provide new data to design potential dosing regimens for future clinical applications that would redefine preventative and treatment approaches against neonatal *E. coli* sepsis.

## Data Summary

The authors confirm that all supporting data and protocols have been provided within the article or through supplementary data files. The nucleotide sequencing data of the iron acquisition genes generated in these analyses are included in Table S1 (available in the online Supplementary Material). The individual accession numbers for isolates are listed in Table S2.

## Introduction

Neonatal early-onset sepsis (EOS) and meningitis are major causes of neonatal mortality worldwide [1]. Every year, an estimated three million cases of neonatal sepsis occur globally, resulting in up to 570,000 neonatal deaths [2]. Worldwide, Gram-negative bacteria predominate as a cause of EOS [3], in contrast to Group B *Streptococcus* (GBS) infections, which have historically been identified as the most common cause of EOS and early-onset meningitis in the USA [4]. However, neonatal *Escherichia coli* infections causing EOS and early-onset meningitis in the USA have increased in the past decade [5], especially among those born pre-term [6]. The increase in *E. coli* EOS has also been documented in other countries such as Italy and Mexico [7,8]. Furthermore, EOS caused by *E. coli* is associated with greater mortality (32% of deaths) than EOS caused by GBS (6%) [5].

Antibiotic resistance in invasive neonatal *E. coli* strains is on the rise. The traditional empirical treatment for EOS recommended by the American Academy of Pediatrics is a combination of both ampicillin and gentamicin [9]. However, a study of 117,484 infants admitted to NICUs at hospitals across the USA from 2009 to 2017 showed that only 32% of *E. coli* isolates causing EOS were susceptible to both ampicillin and gentamicin, and 10% were non-susceptible to both antibiotics [10].

Maternal intrapartum antibiotic prophylaxis (IAP) is effective in reducing the incidence of GBS EOS [5]. However, 82% of *E. coli* isolates that caused sepsis in neonates whose mother received ampicillin for IAP were resistant to ampicillin [5]. These data demonstrate that current IAP is not a reliable measure to prevent *E. coli* neonatal invasive disease. The widespread antibiotic resistance and the lack of effective measures to prevent neonatal *E. coli* sepsis underscore the urgent need to develop novel therapeutic approaches and preventative interventions against this life-threatening disease in newborns.

Lactoferrin (LF) is a naturally occurring prebiotic that has been investigated as an intervention against sepsis. LF is a cationic glycoprotein found predominantly in milk, and it belongs to the transferrin family of iron-binding proteins [11]. The major antimicrobial mechanism of LF is attributed to its capacity to sequester iron, which is crucial for microbial growth and for the pathogenicity properties of invasive *E. coli*. Invasive *E. coli* isolates that cause disease outside the intestinal tract are referred to as extraintestinal pathogenic *E. coli* (ExPEC) and include strains that cause sepsis and meningitis in newborns [12]. ExPEC strains possess multiple virulence factors such as iron acquisition mechanisms, including siderophores and other specialized iron transport proteins, which are relevant to their ability to cause invasive disease [13]. LF reversibly binds ferric iron and siderophore-bound iron secreted by pathogenic bacteria [11,14]. In addition to iron sequestration, several other antimicrobial effects of LF have been described in detail [15]. LF binds to bacterial LPS, which results in LPS neutralization and *E. coli* cell membrane destabilization [16]. LF has also been shown to decrease biofilm formation and to block bacterial adhesion to eukaryotic cells, thus interfering with colonization and invasion [17]. Interestingly, LF also plays a role in modulating the microbiome. LF counteracts dysbiosis by indirectly promoting the growth of beneficial bacteria, such as *Lactobacillus* spp., in the gastrointestinal and female genitourinary tracts [18]. Lactobacilli species then produce bacteriocins that prevent pathogenic bacterial growth [19].

Clinical studies have examined the immunomodulatory and antibacterial effects of LF in preventing neonatal sepsis [20]. LF has been shown in clinical trials to decrease late-onset sepsis in human neonates [21,22]. In addition to studies in humans, pre-clinical animal studies demonstrated increased survival and decreased multi-organ bacterial dissemination in rat pups that were inoculated with *E. coli* and were subsequently fed LF [23]. However, studies that examine LF’s antibacterial effects on various *E. coli* strains specifically associated with neonatal sepsis are lacking.

Preliminary data from our laboratory have shown that human lactoferrin (hLF) is effective in impairing *in vitro* growth of the well-characterized archetypal neonatal invasive *E. coli* strain RS218 that was isolated in the 1970s and known for its ability to cause severe neonatal infections including sepsis and meningitis [24,25]. Moreover, we showed that maternal administration of hLF to pregnant mice led to a marked reduction of invasive RS218 infection in the embryos [26]. However, hLF is expensive and difficult to isolate compared to bovine LF (bLF), which is available in industrial quantities at much lower cost, therefore representing a more accessible alternative [27]. bLF shares 77% amino acid homology with hLF, and *in vitro* studies have demonstrated the effective antimicrobial activity of bLF against non-neonatal *E. coli* strains [15,28]. Clinical studies demonstrating LF’s protective effects against neonatal infections have been done predominantly with bLF, which has been deemed safe in this population [29]. However, the specific antimicrobial effects of bLF against a variety of *E. coli* strains that cause neonatal sepsis in humans have not been demonstrated, limiting the understanding of its utility as a strategy against neonatal *E. coli* invasive infections.

The objective of this project was to determine the effects of increasing concentrations of bLF on the *in vitro* growth of a variety of representative neonatal septicaemia-causing *E. coli* isolates. These neonatal ExPEC strains were selected based on their clinical relevance, genetic diversity and virulence potential. ExPEC isolates belong to common multi-locus sequence types (MLST) that cause invasive diseases in humans. Multi-locus sequence typing is a genotyping method used to study the relatedness of *E. coli* strains and their evolution [30]. Moreover, certain sequence types (STs) are recognized for their high pathogenicity properties and greater content of virulence factors. For example, ST131 is responsible for many instances of *E. coli* bacteraemia in both children and adults [31]. We and others described the initial MLST-based molecular epidemiology of contemporary neonatal invasive *E. coli* strains in the USA, highlighting the predominance of ST131, as well as ST95 and ST69 [32,34]. ST131 and ST95 strains belong to *E. coli* phylogroup B2, whereas ST69 strains belong to phylogroup D, both predominant phylogroups among *E. coli* causing invasive disease in humans [35]. For this study, we specifically selected neonatal *E. coli* septicaemia clinical isolates belonging to ST131, ST95 and ST69. We hypothesized that the growth of these *E. coli* isolates that cause septicaemia in newborns would decrease in the presence of increasing concentrations of bLF. Additionally, we anticipated that iron acquisition virulence genes would be prevalent among these strains, given their role in the pathogenesis of invasive infections caused by ExPEC strains.

## Methods

### Neonatal *E. coli* strains

The clinical *E. coli* strains included in this study were isolated from newborns with septicaemia, which we have characterized in regards to the *E. coli* phylogroups they belong to [33]. Among these invasive neonatal *E. coli* strains, our studies showed that strains that belonged to phylogroup B2 dominated, followed by strains included in phylogroup D [33]. Phylogroup B2 is characterized by *E. coli* isolates commonly bearing the K1 capsule, an important virulence factor that protects bacteria against phagocytosis and is prevalent in *E. coli* strains that cause neonatal septicaemia and meningitis and that was prevalent in the neonatal *E. coli* strains we have studied in the past [33, 36].

We have also categorized neonatal *E. coli* sepsis isolates into MLSTs, which define specific clonal groups and virulence characteristics among *E. coli* strains [31]. STs 95 and 131 were the most prevalent among the invasive neonatal *E. coli* strains we have studied [33,37]. ST95, ST131 and ST69 *E. coli* strains were also dominant ExPEC STs among invasive neonatal strains in other studies [31,34].

In the present study, we studied a total of six invasive neonatal *E. coli* clinical isolates, five from our collection, and also the archetypal neonatal invasive *E. coli* isolate RS218, which belongs to ST95 [25]. Of the five contemporary neonatal *E. coli* septicaemia strains we included herein from our collection, we chose strain SCB12 as a representative of ST95 strains for this study, with observed antibiotic susceptibility and molecular characterization shown in Table 1. SCB31 and SCB58 were two strains that belonged to ST131 included for this study. SCB29 and SC61 were used to represent the ST69 strains [37]. The above strains were obtained from the clinical microbiology laboratory with approval by the local Institutional Review Board as we previously described [33]. The archetypal neonatal meningitis-causing RS218 strain, which belongs to phylogroup B2 and ST95, and bears the K1 capsule, was also included as a reference strain [32,38]. All contemporary strains showed resistance to ampicillin and variable susceptibility to gentamicin, tobramycin, ciprofloxacin and trimethoprim-sulfamethoxazole (Table 1). Of note, strain SCB58 is a multidrug-resistant isolate per the definition by Sievert *et al*. [39].

**Table 1.** *E. coli* isolates from newborns with septicaemia selected for investigation

*E. coli* isolate	Antibiotic susceptibility					Phylogroup	MLST Classification
	Amp	Gen	Tobra	Cipro	T/S		
**RS218**	S	S	S	S	S	B2	ST95
**SCB12**	R	S	S	S	S	B2	ST95
**SCB29**	R	R	R	S	R	D	ST69
**SCB31**	R	R	I	S	S	B2	ST131
**SCB58**	R	S	R	R	R	B2	ST131
**SCB61**	R	S	S	S	S	D	ST69

Data adapted from Cole *et al*. [33] and Farahbakhsh *et al*. [37].

Amp, ampicillin; Cipro, ciprofloxacin; Gen, gentamicin; I, intermediate resistance; R, resistant; S, susceptible; Tobra, tobramycin; T/S, trimethoprim/sulfamethoxazole.

### Bacterial growth comparisons by OD measurements

To determine the antibacterial effects of bLF against neonatal *E. coli* isolates, we performed growth curves by measuring OD at regular intervals over time. This method generates accurate and reproducible data to quantify and compare bacterial growth under various testing conditions [40,41]. Prior to testing, all bacterial strains were kept frozen at −80 °C in glycerol stocks. For these experiments, each strain was streaked onto Luria–Bertani (LB) agar plates and grown overnight at 37 °C. A single colony of each isolate was inoculated into LB broth and grown in an orbital incubator shaker at 250 r.p.m. and maintained at 37 °C for 6 h. Bacterial suspensions were then diluted to a final concentration of 5×10^5^ c.f.u. ml^−1^ in sterile LB medium.

The antibacterial effects of bLF versus hLF have been compared in diarrhoea-producing enteropathogenic *E. coli* strains, for which bLF showed a lesser antibacterial effect. However, bLF had a stronger effect on promoting intestinal differentiation and apoptosis compared with hLF [42]. The *in vitro* antibacterial effect of bLF specifically against various neonatal septicaemia strains (which are Extra-intestinal Pathogenic *E. coli*, or ExPEC strains) has not been studied. Therefore, this study was performed to address this important question. For this, bLF (Hilmar Ingredients^®^, Hilmar, CA, USA) was suspended in 7.2 pH phosphate buffer solution to a concentration of 50 mg ml^−1^. This solution was sterilized by filtering through a 0.22 µM filter. This working concentration of 50 mg ml^−1^ bLF was further diluted to a final concentration of 0.1, 1.0 and 10 mg ml^−1^ into LB containing the bacterial suspensions. Bacterial suspensions in LB broth without LF (0 mg ml^−1^) were included for comparisons. The mean (sd) pH of LB broth after addition of bLF was as follows: 0 mg ml^−1^ bLF, pH=6.83 (±0.3); 0.1 mg ml^−1^, pH=6.9 (±0.01); 1 mg ml^−1^, pH=6.88 (±0.05); 10 mg ml^−1^, pH=6.89 (±0.01). LB-only wells were also included to ensure the absence of contamination. The non-pathogenic *E. coli* DH5α strain was used as a control. Two hundred microlitres of these solutions were added per well to a 96-well plate in quadruplicates. A BMG Labtech^®^ (BMG, NC, USA) ClarioSTAR Plus microplate reader was used to measure OD at 600 nm at 30 min intervals over 20 h at 37 °C.

Experiments were repeated at least three times with 8–12 replicate wells per strain per each bLF concentration. The Shapiro–Wilk test was used to determine the normality of OD data. OD area under the curve (AUC) values of growth curves were calculated using the trapezoidal method as previously described [43,44]. Normally distributed data were analysed with ANOVA, and multiple comparisons were done with Tukey’s test. Nonparametric data were analysed with Kruskal–Wallis tests for multiple comparisons that were analysed with Dunn’s test. For all tests, a *P* value<0.05 was considered statistically significant. Statistical analysis was done using IBM SPSS^®^ v 29.0 (IBM Corp, Armonk, NY) and with GraphPad Prism^®^ v 10.5.0 (Dotmatics, Boston, MA).

### Whole-genome sequencing and identification of iron acquisition genes

To investigate the prevalence of iron acquisition virulence genes in the strains included in the study, whole-genome sequencing (WGS) was obtained on all *E. coli* isolates using previously established protocols [45]. Briefly, genomic DNA was purified with the Qiagen^®^ DNeasy extraction kit. DNA libraries were then prepared and sequenced using Illumina HiSeq technology. Then, obtained sequences were assembled and analysed to determine the presence of iron acquisition virulence genes. This was done using the VirulenceFinder 2.0 database available at the Center for Genomic Epidemiology from the Technical University of Denmark [46]. The database includes genes associated with iron acquisition that are characteristic of ExPEC strains. These genes are involved in ExPEC’s ability to produce invasive infections by facilitating the bacteria to scavenge iron from the host. The iron acquisition virulence genes included *chuA* (outer membrane hemin receptor), siderophore-related genes (*fyuA*, *iroN*, *irp2*, *iucC* and *iutA*) and the iron transport protein gene *sitA* [47]. Parameters for gene identification were set to a threshold identity of 90%, and a minimum sequence length of 60% of each reference gene in the database.

## Results

**Fig. 1.**
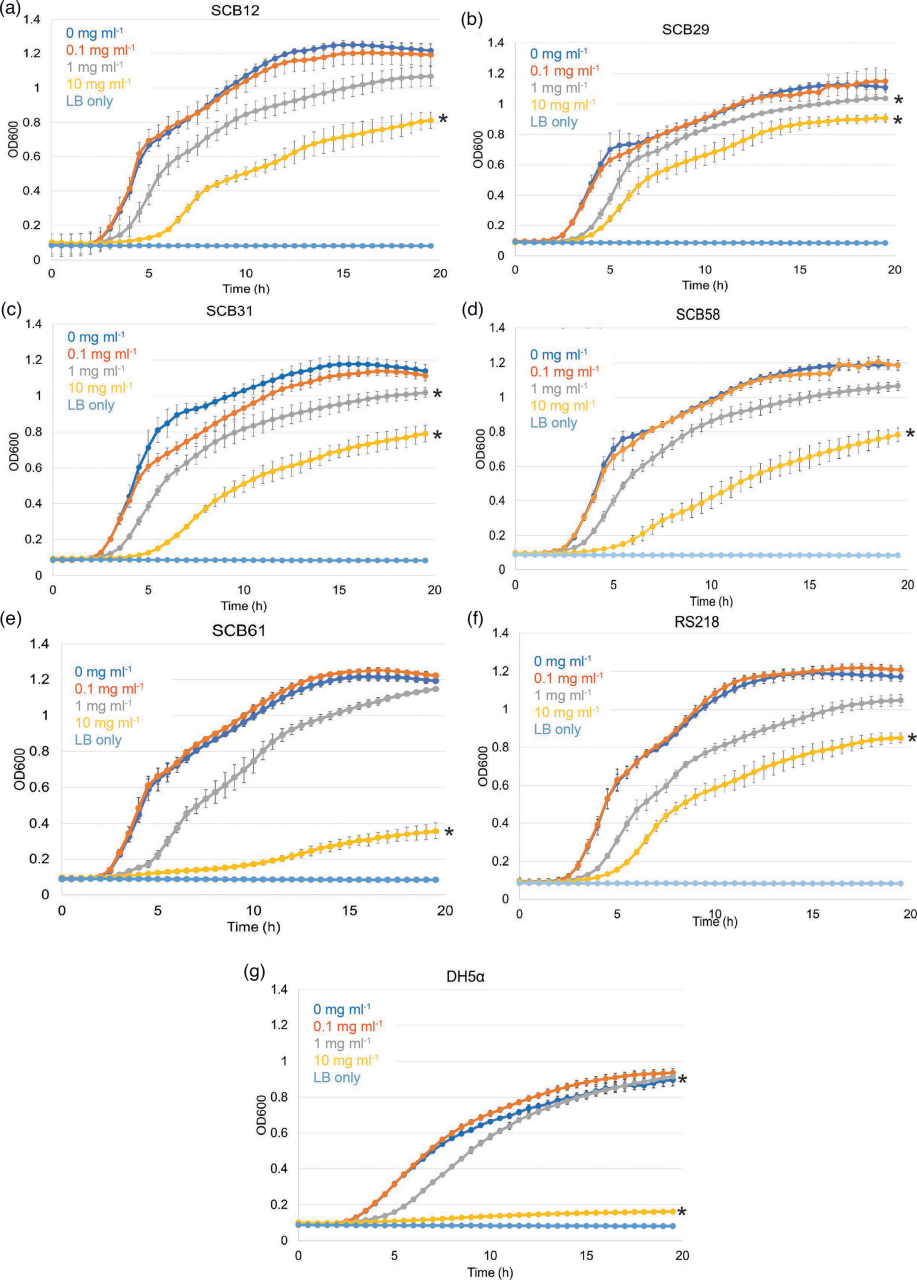
bLF impairs *in vitro* growth of neonatal septicaemia-causing *E. coli* strains in a dose-dependent fashion. Growth curves of neonatal septicaemia *E. coli* strains SCB12 (a), SCB29 (b), SCB31 (c), SCB58 (d) and SCB61 (e), with archetypal invasive neonatal septicaemia *E. coli* strain RS218 (f) and non-pathogenic strain DH5α (g) included as controls. Asterisks (*) indicate a statistically significant difference between the indicated condition and the 0 mg ml^−1^ bLF condition. Experiments were repeated at least three times with 8–12 replicate wells per strain per each bLF concentration (*P*<0.05).

To test our hypothesis regarding the antibacterial effect of bLF against neonatal *E. coli* isolates, we performed growth curves of all *E. coli* isolates exposed to increasing concentrations of bLF. The growth curves generated under the various conditions tested and with data recorded over 20 h for each individual strain are shown in Fig. 1. We observed a highly significant decrease in growth for all strains in the presence of 10 mg ml^−1^ bLF. Fig. 1 shows the significant decrease in growth at the 10 mg ml^−1^ bLF concentration for the five contemporary clinical *E. coli* invasive isolates tested: SCB12, SCB29, SCB31, SCB58 and SCB61, as well as for the archetypal invasive neonatal strain RS218, and the non-pathogenic DH5α strain. Growth at the 1 mg ml^−1^ bLF condition was also significantly reduced in strains SCB29, SCB31 and DH5α.

**Fig. 2.**
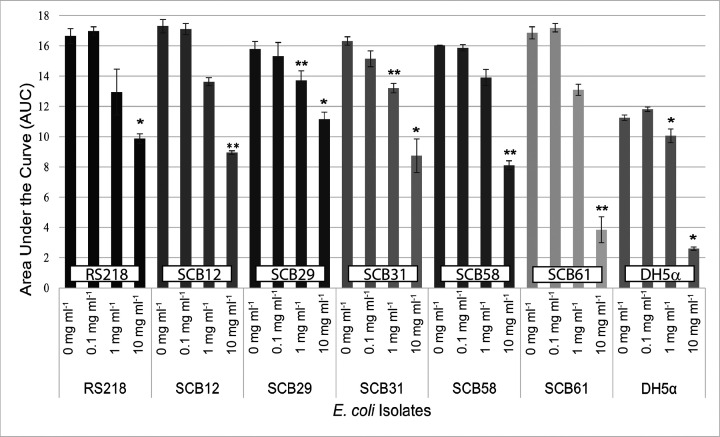
Comparisons of AUC bacterial growth data among all *E. coli* strains. OD AUC values of neonatal septicaemia *E. coli* isolates grown in the presence of increasing concentrations of bLF. DH5α is a non-pathogenic control strain. Bars depict means and sd for each condition tested. Asterisks indicate statistically significant differences compared to the 0 mg ml^−1^ bLF condition for each individual strain. Experiments were repeated at least three times with 8–12 replicate wells per strain per each bLF concentration (*P<0.01, **P<0.05).

In addition to investigating the bacterial growth kinetics by comparing the growth curves generated with individual OD measurement at each time point, the total AUC was also calculated for all conditions tested, and their comparisons are shown in Fig. 2. All strains demonstrated significant growth decrease when exposed to bLF as compared with growth without bLF as shown by AUC comparisons. The lowest AUC values were obtained with the 10 mg ml^−1^ bLF concentration for all strains. Taken together, these data demonstrate that the antibacterial effect of bLF is consistent across this representative panel of neonatal *E. coli* invasive strains and that the antibacterial effect was most pronounced at the highest concentration of bLF tested.

Next, we generated and analysed bacterial WGS data to assess the prevalence and distribution of relevant iron acquisition virulence genes known to play a critical role in *E. coli* pathogenesis. We hypothesized that iron acquisition virulence genes would be prevalent among the neonatal invasive *E. coli* strains we studied, given the role of these iron acquisition genes in the pathogenesis of invasive infections caused by ExPEC strains. The iron acquisition virulence genes found in each strain are shown in Table 2. Seven iron acquisition virulence genes with unique functions were identified: the TonB-dependent outer membrane hemin receptor *chuA*, yersiniabactin siderophore receptor *fyuA*, siderophore receptor *iroN*, high-molecular-weight protein 2 nonribosomal peptide synthetase *irp2*, the aerobactin siderophore synthetase *iucC*, ferric aerobactin receptor *iutA* and the iron transport protein *sitA*. All the pathogenic strains included in this study carried various iron acquisition virulence genes. The two neonatal ST95 strains, RS218 and SCB12, carry *chuA*, *fyuA*, *iroN*, *irp2* and *sitA*. The neonatal isolates SCB29, SCB31 and SCB58 carried the greatest number of iron acquisition virulence genes. In contrast, SCB61 had the fewest iron acquisition genes among the neonatal strains. Sequence identity for the genes identified was high, ranging from 99.8% to 100%. Sequence length alignment was 100% for all genes found except for *irp2* in SCB31, which had a matching length of 89%. Importantly, the non-pathogenic laboratory strain DH5α did not contain any of the iron acquisition virulence genes. The hit in genome sequences within all isolates that matched iron virulence genes included in the VirulenceFinder 2.0 database is shown in Table S1.

**Table 2.** Presence of iron acquisition virulence genes determined by whole-genome sequencing analysis of neonatal *E. coli* septicaemia isolates

*E. coli* strain	Phylogroup	ST	Iron acquisition virulence genes						
			*chuA*	*fyuA*	*iroN*	*Irp2*	*iucC*	*iutA*	*sitA*
RS218	B2	95							
SCB12	B2	95							
SCB29	D	69							
SCB31	B2	131							
SCB58*	B2	131							
SCB61	D	69							
DH5α	A	1060							

* Multi-drug-resistant isolate.

Detected genes are indicated in black cells. White cells indicate absence of gene.

chuA, outer membrane hemin receptor; fyuA, siderophore receptor (yersiniabactin); iroN, siderophore receptor; irp2, high-molecular-weight protein 2 nonribosomal peptide synthetase; iucC, aerobactin siderophore synthetase; iutA, ferric aerobactin siderophore receptor; sitA, iron transport protein; ST, sequence type.

**Fig. 3.**
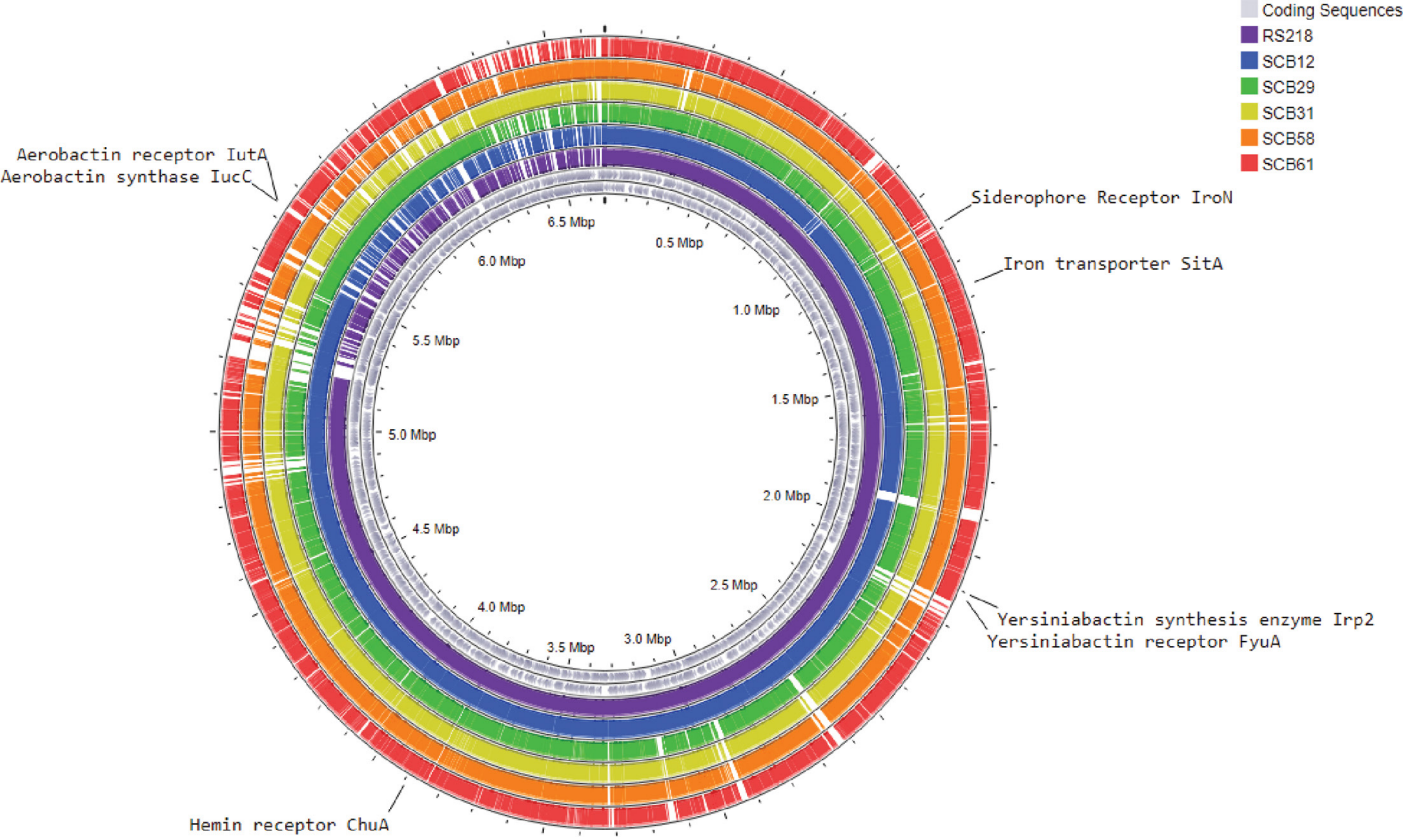
Pangenome analysis of iron acquisition virulence genes demonstrates their presence in all pathogenic neonatal *E. coli* septicaemia isolates. The two innermost gray circles represent all coding sequences among the strains. Each additional circle represents individual whole genome sequences. Most iron acquisition virulence genes were present in all strains. However, *iroN* was only found in RS218 and SCB12. The aerobactin receptor and synthase genes were only present in SCB29, SCB31 and SCB58. Graph was generated using Proksee [61].

To further understand the relationship of the iron acquisition virulence genes identified among the sequenced *E. coli* genomes, we performed a pangenome analysis using Gview using the methods we previously described [45]. This approach allowed detailed visualization of shared unique genomic regions among the strains. For these comparisons, RS218 was used as the reference strain, and the rest of the strains were compared by performing Basic Local Alignment Search Tool analysis [48]. Fig. 3 shows a circular graphical representation of the pangenome and highlights the presence or absence of each individual iron acquisition gene within each isolate sequence. Additionally, we examined genes located adjacent to each iron acquisition virulence gene. These neighbouring genes and their genomic arrangements are shown in Fig. S1.

## Discussion

The results of this study demonstrate that bLF impairs *in vitro* growth of neonatal septicaemia-causing *E. coli* isolates, including isolates resistant to typical antibiotics used to empirically treat and prevent neonatal sepsis. This growth impairment is greater at increasing concentrations of bLF and is maintained regardless of the presence of iron acquisition virulence genes in these isolates.

Other studies have demonstrated an inhibitory effect of hLF on the growth of various pathogenic *E. coli* strains such as enterohaemorrhagic O157:H7 isolates, and enteropathogenic and enterotoxigenic *E. coli* strains, which possess different virulence factors and produce disease via different pathogenic mechanisms than those used by ExPEC strains that produce neonatal septicaemia [49,52]. However, to our knowledge, our study is the first to examine the effects of bLF, a much more accessible protein, on the *in vitro* growth of *E. coli* (ExPEC) isolates causing neonatal sepsis, including those resistant to multiple antibiotics. Therefore, these results support the continued use of bLF in newborns in clinical studies evaluating its use for the prevention of sepsis, which to date have only included testing of bLF, given its greater availability as compared to hLF and known safety profile [22]. Moreover, bLF-derived peptides have shown greater antibacterial activity as compared to peptides derived from hLF [14]. The consistent response to bLF we observed across the representative strains we studied, regardless of whether they showed an antibiotic-resistant phenotype to multiple antibiotics or a more susceptible profile, underscores the potential of broad-spectrum antimicrobial efficacy of bLF. Thus, our results support the consideration of bLF as an intervention against *E. coli* neonatal sepsis.

bLF administered enterally has been effective to prevent late-onset sepsis in newborns, but doses have been variable, and a dose range that is safe and effective has not been clearly established [22]. The LF concentrations we tested are within those physiologically present in human colostrum (around 3–9 mg ml^−1^) and mature milk (1–3 mg ml^−1^) [53,55]. bLF has an excellent safety profile in neonates [20]; bLF doses up to 300 mg kg^−1^ body weight per day are safe for consumption by preterm neonates [56]. Our results demonstrating growth impairment of a variety of neonatal *E. coli* isolates at the concentrations we evaluated provide relevant information that could be applied to further define potential dosing regimens, which will also necessitate consideration of other factors such as intestinal degradation and absorption that are relevant for *in vivo* translation. Additional *in vitro* data, as well as clinical studies, will be needed to determine optimal doses that will be safe and effective to prevent invasive infection by antibiotic-resistant organisms, particularly *E. coli*. This information is particularly relevant, given that the effective therapeutic levels of LF in newborns for any indication have not yet been defined [56].

Iron acquisition is crucial for the virulence properties of ExPEC strains, including those causing invasive disease in neonates [57]. The effect of LF on bacterial growth of various clinical ExPEC strains causing neonatal septicaemia had not been investigated. Our study demonstrates that despite the presence of various iron acquisition genes, LF inhibited the growth of these invasive strains. Moreover, bLF showed the greatest inhibitory effect against *E. coli* DH5α, likely at least in part due to the lack of iron acquisition virulence genes in this non-pathogenic strain. Future studies are needed to further elucidate the specific mechanisms involved in the antibacterial effects of bLF against *E. coli* strains that cause invasive disease in newborns.

One limitation of this study is the testing of a small sample of neonatal *E. coli* invasive isolates. However, these isolates are representative of *E. coli* strains that cause severe infections in newborns, and our findings demonstrating their susceptibility to increasing concentrations of bLF provide new relevant information, given that previous studies, including those testing hLF against neonatal invasive *E. coli*, have only been done in fewer neonatal *E. coli* strains [24,25]. Future studies will examine bLF’s effects on bacterial growth of other neonatal sepsis-causing *E. coli* isolates. Another limitation is that the expression of the iron acquisition virulence genes prevalent in the neonatal strains was not confirmed. The functional activity of these iron acquisition genes is influenced by variable growth conditions both *in vitro* and *in vivo*. For example, others have found that expression of *iutA* in invasive ExPEC strains grown in LB broth is more pronounced in stationary phase, and even greater during infection in animals [58]. Greater expression of *iroN* compared with *iutA* has also been documented in other ExPEC strains during *in vitro* growth in LB broth [59]. These studies have also shown that expression of these genes increases in low-iron environments; therefore, gene expression is context-dependent, and gene presence is not synonymous with resistance to bLF. It is possible that bLF’s growth inhibition might differ in other conditions, such as in low-iron environments like neonatal serum, although we speculate bLF’s antibacterial activity to persist, not only through its iron-sequestering properties but also via its additional antibacterial properties such as LPS binding, which destabilizes the bacterial outer membrane increasing its permeability [60]. Additional studies are needed to evaluate how bLF influences virulence mechanisms linked to the function of iron acquisition genes and their role in the pathogenesis of *E. coli* isolates that cause neonatal sepsis. Despite these limitations, our studies provide evidence for the first time of the antibacterial effect of bLF on neonatal ExPEC isolates that carry iron acquisition genes relevant to virulence.

Finally, bLF has many demonstrable effects on living systems besides its ability to inhibit microbial growth. The findings we present are strictly generated *in vitro*, and host factors, immune modulation and gastrointestinal environment could alter efficacy. Nevertheless, the data generated in this study are relevant to our planned *in vivo* studies, which will examine additional antibacterial and immunomodulatory effects against *E. coli* strains that cause neonatal sepsis in living systems. These animal studies will also provide additional information on the safety and efficacy of bLF as an intervention against neonatal *E. coli* invasive infections.

## Conclusion

Our results confirm our hypothesis that bLF impairs the *in vitro* growth of representative neonatal septicaemia-causing *E. coli* isolates in a concentration-dependent manner. The inhibitory effect on growth of neonatal *E. coli* strains is consistent regardless of the presence of iron acquisition genes known to contribute to virulence. These data underscore the protective role that LF has against severe *E. coli* infections in newborns.

## Supplementary material

10.1099/jmm.0.002116 Uncited Supplementary Material 1.

10.1099/jmm.0.002116 Uncited Supplementary Material 2.

## Abbreviations

AUC: area under the curve
bLF: bovine lactoferrin
ExPEC: extraintestinal pathogenic *Escherichia coli*
GBS: group B *Streptococcus*
IAP: intrapartum antibiotic prophylaxis
LB: Luria–Bertani
LF: lactoferrin
STs: sequence types
WGS: whole-genome sequencing
