# Supplementary material for: Bovine lactoferrin maintains antibacterial effect against neonatal Escherichia coli septicaemia isolates despite the presence of iron acquisition genes
Source: J Med Microbiol. 2026 Jan 21;75(1):002116. doi: 10.1099/jmm.0.002116 (PMC12825036; doi:10.1099/jmm.0.002116)

Supplemental Figure 1. Genes associated to iron-acquisition virulence genes by pangenome analysis of neonatal *E. coli* septicemia isolates.

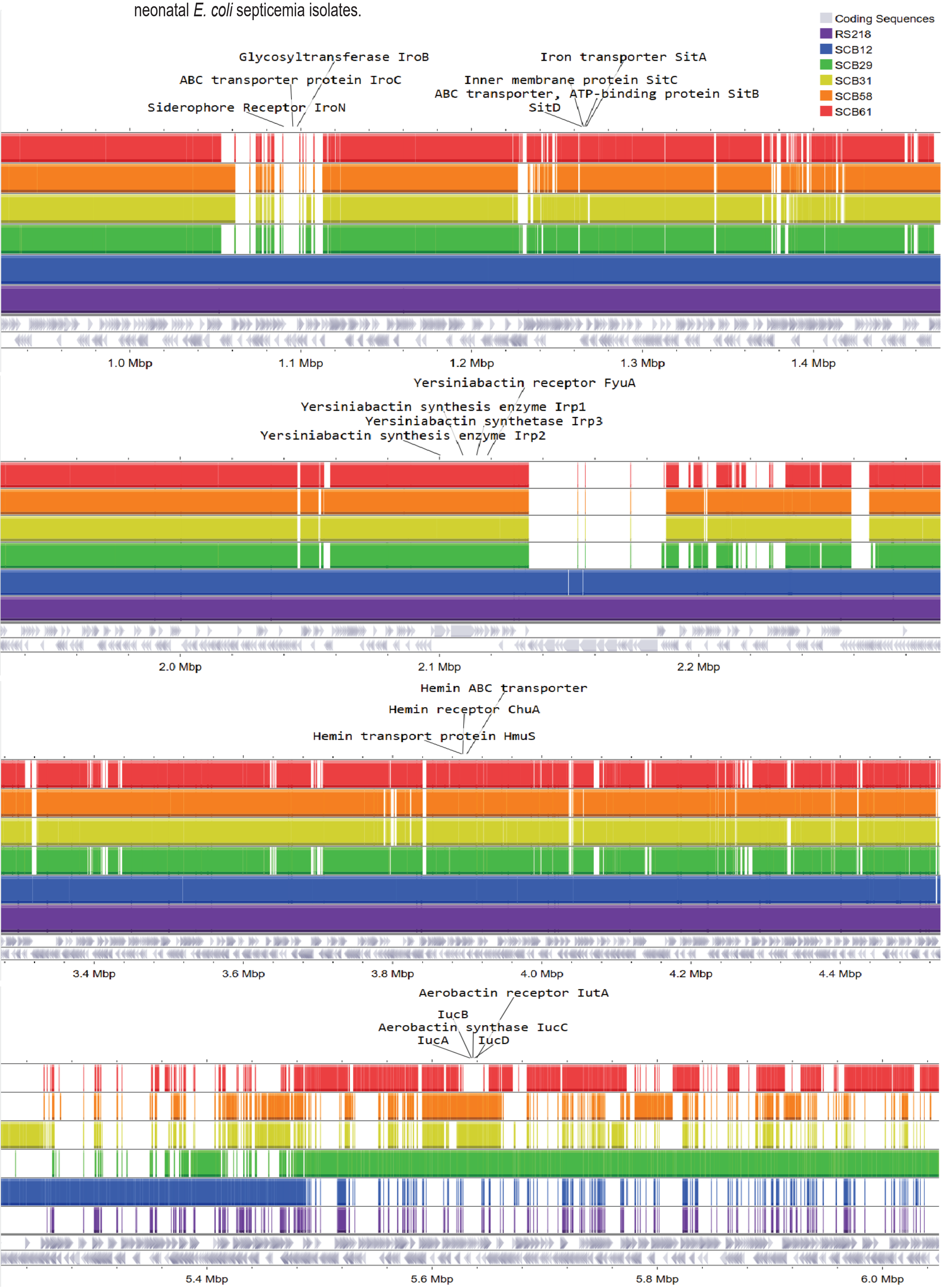

Supplement: Uncited Supplementary Material 1. [file jmm-75-02116-s001.pdf]
